# Transgenerational Developmental Programming of Ovarian Reserve

**DOI:** 10.1038/srep16175

**Published:** 2015-11-03

**Authors:** C. E. Aiken, J. L. Tarry-Adkins, S. E. Ozanne

**Affiliations:** 1 University of Cambridge Metabolic Research Laboratories, Institute of Metabolic Science, Addenbrooke’s Hospital, Cambridge CB2 0QQ, United Kingdom; 2 Department of Obstetrics and Gynaecology, University of Cambridge, Box 223, The Rosie Hospital and NIHR Cambridge Comprehensive Biomedical Research Centre, Cambridge CB2 0SW, United Kingdom.

## Abstract

Exposure to an adverse early-life environment leads to long-term health problems, many of which are recapitulated in subsequent generations. The female reproductive tract is particularly sensitive to early-life influences, and plays a pivotal role in programming the conceptus. We examine the influence of suboptimal grandmaternal diet on reproductive potential of granddaughters in the absence of any further dietary manipulations in the daughters in a rat low-protein diet model. Exposure to low-protein grand-maternal diet leads to decreased ovarian reserve and increased intra-abdominal fat mass in granddaughters, accompanied by accelerated accumulation of oxidative stress and mtDNA copy number instability in the ovaries. Ovarian telomere length declines more rapidly in the exposed granddaughters, indicating accelerated ageing in the reproductive tract. Thus, we demonstrate that suboptimal grandmaternal diet during pregnancy accelerates reproductive ageing across subsequent generations. These findings have important implications for understanding both individual rates of decline in fertility with age, and the clinical impact of current global trends towards delayed childbearing.

The early life environment plays a crucial role in determining health outcomes in later life^1^. Exposure to a sub-optimal environment during development leads to changes in physiology and metabolism that persist into adulthood, and which may be recapitulated in further generations. The transmission of developmentally programmed phenotypes across generations has been observed in both human cohorts and animal models^2^,^3^,^4^.

Factors regulating rates of reproductive ageing in females are currently poorly understood. Particularly in light of global trends towards delayed child-bearing^5^ and increasing interest in preserving fertility at later ages, new insights into identifying women who are at risk of an early decline in their reproductive potential would be valuable in clinical practice. Aside from direct effects on fertility, age at menopause has important implications for other aspects of health in later life, including the risks of osteoporosis^6^, ischaemic heart disease^7^ and ovarian cancer^8^. Currently, there are few means available for predicting future reproductive ageing on an individual basis. This study aims to identify whether grandmaternal pregnancy influences later-life reproductive potential in grand-daughters. Identifying transgenerational risk factors for accelerated female reproductive ageing would not only pave the way for counseling high-risk women, but also for the future development of interventions targeting preservation of ovarian reserve in high-risk groups.

Maternal nutrition during pregnancy and lactation has profound effects on the intrauterine environment, leading to developmental programming in a wide range of organs and systems^9^,^10^,^11^, including the reproductive system^12^. Observational studies in humans and other mammals suggest that the development and ageing of the female reproductive tract is particularly vulnerable to the sequelae of an adverse early-life environment induced by maternal diet^13^,^14^,^15^. Aspects of reproductive dysfunction, including dysregulation of estrous cycling^16^, early onset of puberty^17^ and decline in fertility with age^18^, have all been observed in offspring following maternal nutritional interventions (previously reviewed in^12^). In particular, a low-protein maternal diet in the rat model is associated with an early prolongation of estrous cycle length with a concomitant decline in conception rate, suggesting early reproductive ageing^18^. Maternal protein restriction is also associated with a pre-pubertal reduction in ovarian follicular numbers^14^. We have previously demonstrated that rats exposed to an isocaloric low-protein maternal diet *in utero* who undergo rapid postnatal catch up growth (recuperated) show decreased ovarian reserve in adulthood, as assessed by both reduced primordial follicle count and serum anti-Müllerian hormone levels^15^. Decreased ovarian reserve was observed alongside strong evidence of accelerated tissue ageing – increased oxidative stress, mitochondrial DNA (mtDNA) instability and accelerated telomere shortening – in the somatic ovarian tissue of the recuperated animals. Adverse effects of maternal diet were exaggerated in offspring at 6 months compared to 3 months, strongly suggesting that developmental programming may be a factor in age-associated fertility problems.

Although it is well established that developmentally programmed phenotypes may be transmitted to subsequent generations^4^, the female reproductive tract in second-generation offspring has not previously been explored. We hypothesize that development within the programmed reproductive tract of the first generation offspring could adversely influence reproductive tract development in a second generation. Such an effect would provide a mechanism independent of direct germ-line modification for re-propagating a developmental programming phenotype beyond the original two generations exposed either directly or indirectly to the programming stimulus. In this study, the hypothesis that suboptimal grand-maternal nutrition can lead to a phenotype of accelerated reproductive ageing in a second generation of offspring is tested in a previously characterized^15^,^19^,^20^ rat low-protein grand-maternal (F0) diet model of developmental programming, with no further nutritional manipulation in the F1 generation.

## Results

### Bodyweight, organ weights and fasting blood glucose levels

**Figure 1:**
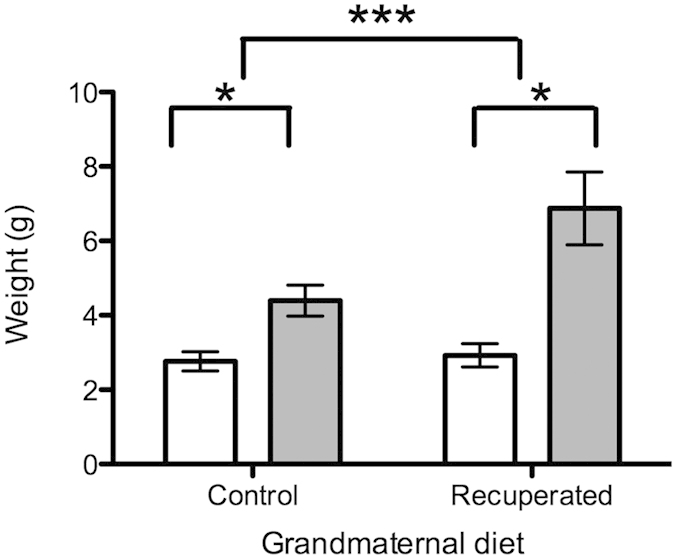
Effect of grandmaternal diet and offspring age on intra-abdominal fat mass. Ovarian fat pads were weighed fresh immediately following dissection. Open bars represent results at 3 months of age, gray bars represent results at 6 months of age. Results are expressed as means (SE); n = 7 per group. *denotes p < 0.05, ***denotes p < 0.001.

There was no effect of grand-maternal diet on offspring body-weight at postnatal day 3 between control and recuperated animals (7.47 ± 0.42 g *versus* 7.15 ± 0.25 g respectively), in contrast to the first generation of offspring, in which low birth weight followed by post-natal catch-up growth was observed^21^. No significant differences were observed in body weight, fasting glucose concentration or in organ weights between control and recuperated groups at either 3 months or 6 months of age (Table 1). At 3 months of age there was no difference in intra-abdominal fat mass between the groups, however by 6 months of age the recuperated group had accumulated significantly more intra-abdominal fat than the control group (p < 0.001) (Fig. 1).

### Primordial follicle counts and serum anti-Müllerian hormone (AMH)

**Figure 2:**
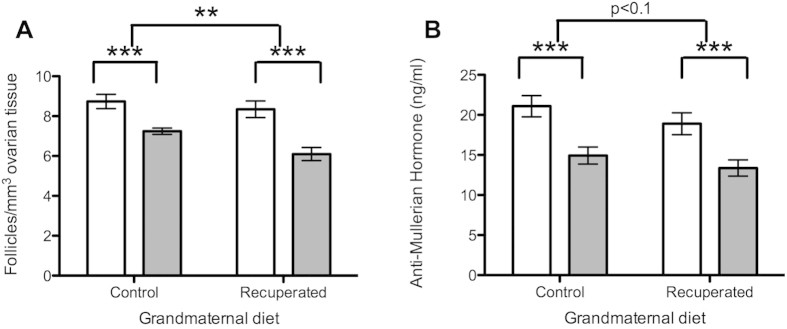
Effect of grandmaternal diet and offspring age on ovarian reserve. Open bars represent results at 3 months of age, gray bars represent results at 6 months of age. **denotes p < 0.01, ***denotes p < 0.001 (**A**) Primordial follicle count per mm^3^ of ovarian tissue in whole fixed ovaries. (**B**) Serum levels of Anti-Müllerian hormone (ng/ml).

Primordial follicle count per mm^3^ of ovarian tissue was not significantly different between control and recuperated offspring at 3 months of age (Fig. 2A). In both groups, the primordial follicle count reduced with age (p < 0.001) as expected, but this was accelerated in the recuperated group compared to the controls (p < 0.01). Serum AMH (produced by granulosa cells, and used here as a proxy measure for antral follicular reserve) decreased in both control and recuperated animals between 3 and 6 months of age, in keeping with an expected decline in antral follicular reserve with age (Fig. 2B; p < 0.001). There was a trend towards lower serum AMH levels in the recuperated group compared to the control (effect of grand-maternal diet p < 0.1).

### Oxidative stress, ageing and anti-oxidant defense mechanisms

**Figure 3:**
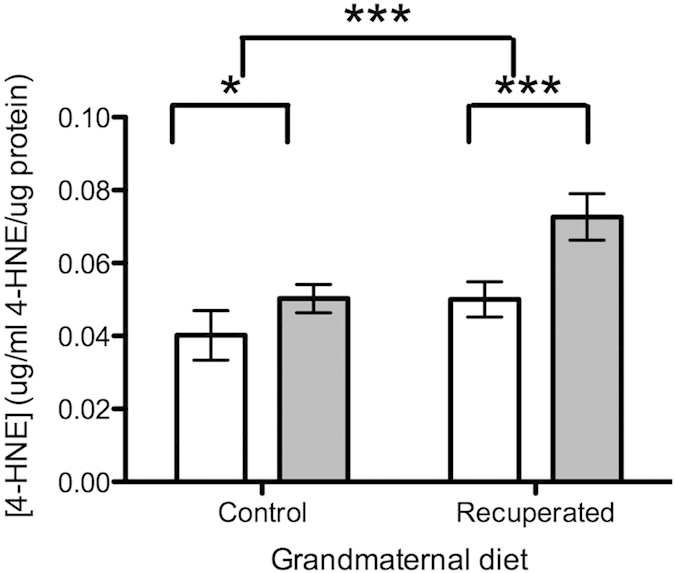
Effect of grandmaternal diet and offspring age on ovarian levels of 4-HNE, reflecting oxidative stress. Open bars represent results at 3 months of age, gray bars represent results at 6 months of age. ***denotes p < 0.001.

Levels of 4-HNE (4-hydroxy-nonenal; a marker of lipid peroxidation, reflecting oxidative stress levels) were significantly increased in the ovarian tissue of the recuperated animals compared to the control group at 6 months of age (p < 0.001) (Fig. 3), however this was unchanged between groups at 3 months of age. Oxidative stress accumulated with age in both grand-maternal diet groups (p < 0.001), but this was exacerbated in the recuperated animals. Expression of a number of cell cycle regulators (p53, p21 and p16^ink^) did not vary with offspring age or with grand-maternal diet (Table 2). Anti-oxidant defense mechanisms were also examined (Table 2). The expression of MnSOD (manganese superoxide dismutase) did not alter between groups either with age or with grand-maternal diet. However, there was reduced expression of CuZnSOD (copper/zinc superoxide dismutase) in the recuperated animals compared to the controls (p < 0.05) at both ages. By contrast, ECSOD (extracellular superoxide dismutase) reduced with age (p < 0.05) but there was no effect of grand-maternal diet on ECSOD expression. Catalase mRNA expression was unaltered by grand-maternal diet or offspring age (Table 2).

### Mitochondrial DNA (MtDNA) copy number and regulation

**Figure 4:**
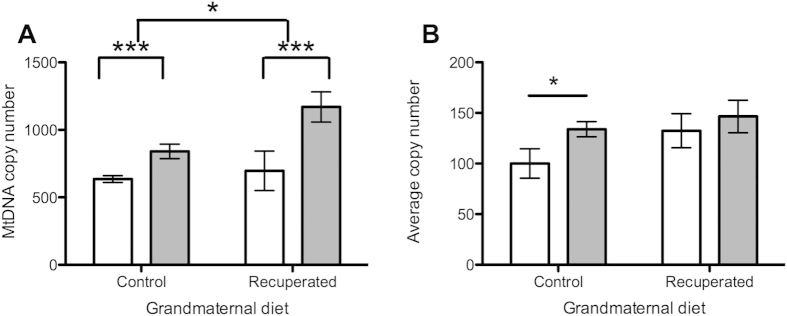
Effect of grandmaternal diet and offspring age on ovarian mtDNA. Open bars represent results at 3 months of age, gray bars represent results at 6 months of age. *denotes p < 0.05, ***denotes p < 0.001 (**A**) MtDNA copy number (ratio to single copy nuclear gene) in ovarian tissue. (**B**) Expression of Tfam (regulator of mtDNA biosynthesis) in ovarian tissue.

At 3 months of age there was no detectable difference in mtDNA copy number between the control and recuperated groups, but by 6 months of age, copy number was significantly elevated in the recuperated group (Fig. 4A). Overall, there was a significant effect of grand-maternal diet on mtDNA copy number (p < 0.05). A strongly significant (p < 0.001) effect of ageing on mtDNA copy number was also observed, with levels increasing between 3 months and 6 months of age in both dietary groups. Expression levels of the master regulator of mitochondrial DNA biogenesis (Transcription factor A, mitochondrial; Tfam) were also measured. Tfam mRNA copy number increased between the 3 and 6-month time points in the control group (p < 0.05), but was already significantly elevated in the recuperated animals by 3 months of age (Fig. 4B).

### Ovarian telomere length

**Figure 5:**
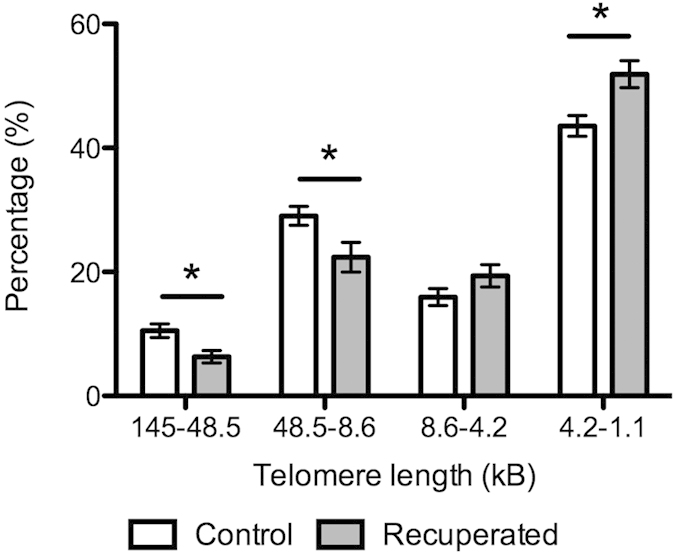
Effect of grandmaternal diet on telomere length at offspring age 6 months. Light grey bars represent control group, dark gray bars represent recuperated group. *denotes p < 0.05.

**Figure 6:**
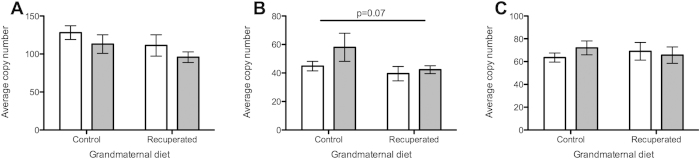
Effect of grandmaternal diet on expression of components of the DNA-activated protein kinase (DNA-PK). Light grey bars represent control group, dark gray bars represent recuperated group. (**A**) Ku70 expression (**B**) Ku80 expression (**C**) DNA-PKcs.

Age-dependent telomere shortening was apparent in both control (p < 0.05) and recuperated (p < 0.05) groups with significantly more short telomeres (1.1–8.6 kB) present at the 6-month time-point. No significant difference in telomere length was observed between control and recuperated animals at 3 months of age. At 6 months of age, there were significantly (p < 0.05) fewer long telomeres (48.5–145 kB) in the recuperated animals compared to the controls (Fig. 5). Conversely there was a higher proportion of short telomeres in the recuperated animals in both of the shorter groups (4.2–8.6 Kb, p < 0.05) and (1.1–4.2 Kb, p < 0.05). There was a highly significant (p < 0.001) interactive effect of age with grand-maternal diet on telomere length at 6 months of age, strongly suggesting accelerated ageing of the ovarian tissue in the recuperated group. Expression of the components of DNA-PK (primarily responsible for repairing double-stranded DNA breaks and hence playing a role in maintaining telomere length) did not vary significantly with offspring age or grand-maternal diet, however there was a trend towards reduced expression of one component (Ku70) in the recuperated group (p = 0.07, Fig. 6).

## Discussion

Reproductive ageing is a topic currently poorly understood, yet of significant interest, particularly in light of global population trends towards delayed childbearing. The insight provided by this study, that early life environment is critical to maintaining reproductive capacity across generations, has important clinical implications at a population level. Ageing of the female reproductive system takes place on an accelerated time-scale compared to other somatic organs, leading to diminished reproductive capacity in the female significantly earlier than senescence in other somatic tissues^22^,^23^, however factors affecting the differing rates of reproductive decline in individuals remain uncertain. We have previously observed that the female offspring of mothers exposed to a suboptimal environment during pregnancy undergo accelerated reproductive tract ageing relative to their control counterparts^21^. In this study we show that a second generation of female offspring, having developed within the first-generation programmed reproductive tract, also have early depletion of their ovarian reserve accompanied by hallmarks of accelerated tissue ageing (increased oxidative stress, mtDNA copy number instability and shortened telomeres).

In common with our findings in the first generation, oxidative stress was significantly increased in the ovarian tissue following exposure to suboptimal grand-maternal diet. In the ovary, increased oxidative stress is recognized to result in decreased follicular reserve and a decline in fertility^24^,^25^. Furthermore, increased oxidative stress is recognized as a final common pathway leading to adverse offspring phenotypes in many developmental programming models^26^,^27^,^28^, however the stress pathways generated by developmental programming stimuli differ between organ systems^29^. MtDNA copy number is highly responsive to oxidative stress, and increases in copy number have been observed in response to stresses including ageing^30^,^31^, endogenous stress *in vivo*^32^,^33^ and developmental programming^15^. Our finding of increased mtDNA copy number in the ovarian tissue of second-generation offspring by 6 months of age is consistent with a phenotype of accelerated ageing in grand-offspring of mothers exposed to low-protein diet in pregnancy.

We observed an accelerated decline in telomere length in the second generation of recuperated grand-offspring compared to their control counterparts. Oxidative stress is known to accelerate telomere shortening^34^, with important consequences for regulating senescence. In the reproductive system, telomere length declines more rapidly than in somatic tissues, which is thought to be the basis for the early decline in female reproductive potential^35^. We previously observed accelerated telomere shortening in conjunction with decreased ovarian reserve in the first generation of recuperated offspring^15^.

Transgenerational effects of developmental programming stimuli have been demonstrated in multiple organ-based and metabolic systems (reviewed in^4^). Here we make the novel finding that the second-generation somatic female reproductive tract is vulnerable to transgenerational programming via the maternal line, despite the lack of any further programming stimulus in the F1 generation. There are two primary mechanisms by which the second-generation offspring may acquire a programmed phenotype – the first is by direct exposure of the germ-line cells to a suboptimal environment F0 early in development, and the second is by development of the conceptus within the programmed F1 reproductive tract. Our results do not allow the two possibilities to be entirely disentangled, and it is possible that both mechanisms make a contribution to the final programmed phenotype. However, our observation that the second generation reproductive tract also shows accumulation of oxidative stress and DNA damage in response to an F0 generation programming intervention is an important finding in establishing the possibility that the third or fourth generation effects observed in developmental programming models^36^,^37^,^38^,^39^ could be a consequence of propagation of the suboptimal phenotype via the reproductive tract environment. This putative mechanism of propagation rather than direct transmission via the germ-line of developmental programming between generations could explain the development of a programmed phenotype even in generations with no direct or indirect exposure to the initial suboptimal environment.

The observation that the trans-generational programming effect in second-generation ovaries is magnified by offspring age highlights the importance of maternal age in determining pregnancy outcomes. In both the second-generation data presented here and our previous work on reproductive tract programming of the first generation of offspring, the adverse effects of the early life stimulus were increased with advancing maternal age^15^. In the second-generation offspring, very little phenotypic change in the ovarian tissue was observed at the 3-month time-point in isolation. Although there are trends in the data to suggest developing adverse effects, none of the measured parameters are significantly different between the control and recuperated groups. However, by mid-reproductive life (6 months of age) a strong phenotype of decreased follicular reserve, oxidative stress and molecular evidence of ageing had developed, which is particularly striking as it occurs well before the age when most rats will begin to display inconsistent estrous cycling. This finding has important potential implications for maximizing reproductive opportunity in populations that have potentially been exposed to adverse environments in earlier generations.

While there is clear evidence of an adverse female reproductive tract phenotype in both the first and second generation of offspring in our low-protein diet model of developmental programming, this is both later in onset and decreased in magnitude in the second-generation of offspring compared to the first^15^. This may represent a lessening of the impact of the initial F0 programming stimulus as it is transmitted through generations, or it may reflect the early age at which the F1 generation were bred within our model. In contrast to the second generation, a phenotype of increased oxidative stress and decreased follicular reserve, with a trend towards telomeric shortening, was already present in the F1 generation offspring by 3 months of age, hence the F1 mothers in this study already had evidence of accelerated ageing in the reproductive tract. However, we hypothesize that the magnitude of the effect on the second generation might be greater as the age of the F1 generation mother increases. This is a key area for future research. Conversely, if the reproductive tract environment rather than germ-line transmission is responsible for the propagation of the second-generation phenotype, then breeding the second-generation females prior to the development of any significant adverse effects in the reproductive tract at 3 months should result in an unimpaired third generation of offspring.

Limitations of the current study include the inability to distinguish between germline modification and re-propagation of the phenotype via development in the programmed reproductive tract from the current experimental data. Future work will focus on disentangling these mechanisms. Unfortunately, due to the small absolute amount of ovarian tissue present in the rat, it was not possible to directly measure protein expression in this model system in addition to performing histological, DNA, ELISA, and extensive gene expression studies. This represents a limitation of the current study. It is a further limitation that we are unable to predict whether the results will be directly applicable in human cohorts.

Our finding that developmental programming effects on the female reproductive tract persist in a second-generation of offspring following a grand-maternal nutritional stimulus has important implications, not only for improved understanding of how ovarian reserve is influenced by the early life environment, but also how programming effects could persist or be recapitulated in future generations. This work establishes the female reproductive tract as a possible key component in trans-generational programming^4^. The finding that an adverse grand-maternal environment leads to molecular evidence of accelerated female reproductive ageing is of particular interest in view of global trends towards older age at first birth^5^. Increasing maternal age is inversely correlated with fertility, however the individual variability in trajectory of declining reproductive function makes future fertility difficult to predict. The finding that ovarian reserve is influenced by dietary interventions in previous generations has clinical implications for fertility counseling and for provision of assisted reproduction technologies. There is a need to validate these findings in epidemiological cohorts in order to quantify the magnitude of the effect in human populations and hence apply these findings to clinical practice. Several promising postnatal interventions have recently been developed to ameliorate the effects of developmental programming in other organ systems^40^, and further work is required to determine whether these may also be useful in maintaining reproductive potential.

## Materials and Methods

### Experimental Design

All animal experiments were approved by the University of Cambridge Animal Welfare and Ethical Review Board. All animal experiments were conducted in accordance with the British Animals (Scientific Procedures) Act (1986). The aim of the study was to develop a transgenerational developmental programming cohort in order to test the hypothesis that suboptimal grandmaternal diet accelerates reproductive ageing in second-generation offspring. The pre-specified outcomes related to reproductive ageing were: primordial follicle counts, serum anti-Mullerian hormone levels, ovarian telomere length, mtDNA copy number and end-products of lipid peroxidation (assayed by 4-HNE levels). Wistar rat dams were fed a standard laboratory chow diet (20% protein) and fed *ad libitum* until pregnancy was confirmed through the observation of vaginal plugs. Pregnant animals were then randomly assigned to a 20% protein diet (control) or an 8% isocaloric low protein (LP) diet, as described previously (18). Both diets were purchased from Arie Blok (Woerden, The Netherlands). Pups born to LP-diet-fed dams were cross-fostered to control-fed mothers at postnatal d3, in order to create recuperated offspring. Each recuperated litter was culled to 4 female pups at random to maximize their plane of nutrition. The control group was the offspring of mothers fed the 20% protein diet during gestation and suckled by 20%-protein-fed dams during lactation. Each control litter was culled to 8 female pups. After weaning, all first-generation offspring were maintained on standard laboratory chow *ad libitum*. At 12 weeks, the female first-generation offspring were mated to produce a second generation of offspring. The second generation was culled using the same protocol as the first generation, suckled by their own mothers and fed *ad libitum* on standard laboratory chow. The model utilized is thus an isocalorific prenatal low protein model that causes *in utero* growth restriction during the index pregnancy, followed by suckling a control fed dam. This mismatch in prenatal and post-natal nutrition promotes accelerated growth and therefore catch-up in body weight in the first generation during lactation. No nutritional interventions were made in the second generation.

Second-generation offspring body weights were recorded at day 3 of life and weekly thereafter. Fasting blood glucose levels were determined using a glucometer (Hemocue, Angelholm, Sweden). At random, one littermate of the second-generation offspring was killed at 12 weeks and the other was killed at 24 weeks of age by carbon dioxide asphyxiation and decapitation. At postmortem, the ovaries, ovarian fat pads and other solid intra-abdominal organs were harvested and weighed fresh, immediately after dissection. One ovary from each animal was snap-frozen in liquid nitrogen and the other fixed in formalin/paraldehyde. The animals utilized in this study were not estrous-staged and hence these results represent a mean effect during the estrous cycle. It is possible that breakdown of results by estrous stage would allow further insight into the effects of oxidative stress on ovarian tissue during the reproductive cycle, but the numbers represented by each group were not powered to allow meaningful subgroup analysis. The fixed ovaries were sectioned and subjected to haematoxylin and eosin (H&E) staining to ensure equal distribution of estrous stages in each experimental group (data not shown). Sample analysis was performed using project codes to blind the investigators to the experimental groups. Eight samples per group were analyzed at each time-point, each sample representing a different litter. The sample size was determined via a power calculation based on the effect sizes seen in our previous study^15^, using an alpha level of 0.05 to give power of 0.8.

### Primordial follicle counts

Fixed ovaries were processed for microscopy and the entire ovary sectioned at 8 μm. Every 6^th^ section was stained with H&E for morphometric analysis (48 μm between analysed sections). Only follicles with a visible oocyte nucleus were counted, in order to avoid repeat counts of the same follicle^41^. Primordial follicles were identified morphologically by the presence of a single layer of flattened granulosa cells surrounding the oocyte^42^. Total volume of each ovary was calculated (section areas  × section thickness  × number of sections) and the follicle count expressed as follicles/mm^3^ of ovarian tissue.

### Anti- Müllerian Hormone measurements

Serum was derived from whole tail-blood samples collected prior to sacrifice. Anti- Müllerian hormone (AMH) was assayed using a rat AMH ELISA kit according to the manufacturer’s instructions (Ansh Labs, Webster, TX, US). The intra-assay co-efficient of variability was 2.84%.

### Oxidative stress analysis

Lipid peroxidation was analyzed using an OxiSelect 4-hydroxy-nonenal (4-HNE) Adduct ELISA kit (Cambridge Biosciences, Cambridge, UK), according to the manufacturers’ instructions.

### Gene expression analysis

RNA was extracted from snap-frozen ovarian tissue using a miRNeasy mini kit (Qiagen, Hilden, Germany) following manufacturers’ instructions, with the addition of a DNaseI digestion step to ensure no genomic DNA contamination. The gene *Myh6* which is not expressed in ovarian tissue was used as a negative control to demonstrate no genomic DNA contamination (all values undetectable). RNA quantification was performed using a NanoDrop spectrophotometer (Nanodrop Technologies, Wilmington, DE, USA). RNA (1 μg) was used to synthesize cDNA using oligo-dT primers and M-MLV reverse transcriptase (Promega). Gene expression was determined using custom designed primers as shown in Table 3 (Sigma, Poole, Dorset, UK) and SYBR Green reagents (Applied Biosystems, Warrington, UK) as previously described^43^. Quantification of gene expression was performed using a Step One Plus RT-PCR machine (Applied Biosystems, Warrington, UK). Equal efficiency of the reverse transcription of RNA from all groups was confirmed through quantification of expression of the house-keeping gene *ppia*. Expression did not differ between groups (effect of maternal diet; p = 0.2, effect of age; p = 0.3 (control 3 m; 110 ± 32, recuperated 3 m; 124 ± 19, control 6 m; 103 ± 24; recuperated 6 m; 115 ± 7; average copy number).

### Telomere length analysis

High-molecular weight DNA was extracted using the DNeasy Blood and Tissue kit (Qiagen, Hilden, Germany) according to the manufacturer’s instructions. DNA quantity and purity was determined using a Nanodrop spectrophotometer (Nanodrop Technologies). Agarose gels were run to ensure all DNA samples were of high-molecular weight. DNA (1.2 μg) was digested with *Hinf*I and *Rsa*I restriction enzymes for 2 h at 37 ^o^C. The restricted samples were quenched with 5x SDS loading buffer (Roche Diagnostics, Mannheim, Germany) and loaded onto agarose gels containing SYBR safe stain (Invitrogen, Paisley, Scotland, UK). After pulsed field gel electrophoresis (PFGE), the gels were checked for non-specific degradation of an undigested DNA control and complete digestion of the enzyme-restricted DNA by visualizing the stained gels under UV light (Syngene, Cambridge, UK). The separated DNA fragments were transferred to nylon membrane (Roche Diagnostics, Mannheim, Germany) by Southern blotting, and telomeric repeat length was determined using a commercial method of chemiluminescent detection as described previously^44^. Molecular weight markers on each gel were a mid-range pulsed-field gel marker (New England Biolabs, Ipswich, MA, USA) and dioxygenin (DIG; low range) molecular-weight marker (Roche Diagnostics, Mannheim, Germany). Standard undigested and digested genomic samples of DNA from a 3-month control animal were also included on each gel to verify digestion efficiency. Telomere signals were analyzed using Adobe Photoshop (Adobe Systems Inc. San Jose, CA, USA) and MacBas software (Fujifilm UK, Bedford, UK). Telomere length was measured as described previously^44^.

### Mitochondrial DNA copy number quantification

Total DNA (mitochondrial and nuclear) was extracted using the DNeasy Blood and Tissue kit (Qiagen, Hilden, Germany) according to the manufacturer’s instructions. A ratiometric assay of the levels of a single-copy mitochondrial gene, *cytochrome c oxidase (Cox1*; primer sequences; Forward 5′-AGACACCTCTCTTTGTATGATCC-3′; Reverse 5′-CGGTCTGTAAGGAGTATAGTGAT-3′), against a single-copy nuclear gene, *nth endonuclease III-like 1 (NTHL1*; primer sequences; Forward 5′-GATTTTGCCTTCCTGTCCATC-3′; Reverse 5′-GAAGCCCAAAACCCTCAGA-3′), was used to estimate the average copy number of mitochondrial DNA (mtDNA)/nuclear DNA (nDNA) (22). Real-time PCR using SYBR Green detection was performed as described previously (32). Standard curves were created using a 2-fold dilution series of a rat genomic DNA standard (Novagen, Darmstadt, Germany). The gradient of the standard curve (*y*) reflects the efficiency of the reaction, with an optimum value of 3.333 (100% efficiency). C_T_ values for *NTHL1* were subtracted from those for *Cox1* to give ΔC_T_. A gradient of ΔC_T_ across the dilution series <0.1 was accepted. Average mtDNA copy number per nuclear genome (2 *NTHL1* copies) was calculated as 2 × 2^(ΔCT)^^45^.

### Statistical Analysis

All data were analyzed using a hierarchical linear model with a random effect for litter and grand-maternal diet and offspring age as fixed effects. This structure accounted for the fact that offspring from a single litter are represented at both 3 months and 6 months of age, and these data cannot be treated as fully independent. Data are represented as means ± SE. A value of P < 0.05 was considered statistically significant. All data analysis was conducted using the R statistical software package version 2.14.1 (R Foundation for Statistical Computing, Vienna, Austria). In all cases, n refers to the number of litters.

## Additional Information

**How to cite this article**: Aiken, C.E. *et al.* Transgenerational Developmental Programming of Ovarian Reserve. *Sci. Rep.* **5**, 16175; doi: 10.1038/srep16175 (2015).

## Figures and Tables

**Table 1:** Effect of grandmaternal low-protein diet (Recuperated) on second-generation offspring postnatal bodyweights, fasting plasma glucose and solid intra-abdominal organ weights.

	Control	6 months	Recuperated	6 months
	3 months		3 months	
Birth-weight (g)	6.7 (1.2)		7.1 (0.25)	
Bodyweight (g)	270 (9)	325 (13)	264 (6)	334 (14)
Fasting blood glucose (mmol/L)	5.5 (0.1)	5.6 (0.5)	5.5 (0.1)	6.6 (0.4)
Ovarian weight (mg)	48 (6)	51 (4)	52 (2)	59 (5)
Kidney weight (mg)	95 (3)	110 (5)	101 (2)	109 (4)
Heart weight (g)	1.1 (0.02)	1.2 (0.06)	1.2 (0.05)	1.2 (0.04)

Results are expressed as means (SE); n = 7 per group.

**Table 2:** Effect of grandmaternal diet on expression of markers of cellular ageing and anti-oxidant defense mechanisms in ovaries.

Gene expression	Control	6 months	Recuperated	6 months	Effect of grandmaternal diet (p value)	Effect of offspring age (p value)
	3 months		3 months			
p53	273 (50)	250 (63)	262 (41)	203 (25)	0.55	0.41
p21	105 (16)	92 (26)	107 (36.3)	90 (21)	0.99	0.57
p16ink	24 (4)	36 (16)	16 (2)	28 (5)	0.28	0.13
MnSOD	502 (46)	486 (57)	538 (66)	554 (51)	0.36	0.99
CuZnSOD	678 (51)	834 (43)	578 (103)	652 (56)	<0.05*	0.11
ECSOD	147 (16)	126 (23)	155 (4)	99 (21.5)	0.61	<0.05*
Catalase	625 (70)	691 (91)	649 (98)	645 (14)	0.56	**0.94**

mRNA expression was measured using RT-PCR. Results are expressed as means (SE); n = 7 per group *denotes p < 0.05.

**Table 3:** Primer sequences for gene expression studies.

Gene	Forward sequence (5′-3′)	Reverse sequence (5′-3′)	Product size (bp)
p53	CCTATCCGGTCAGTTGTTGG	CGTATGAGGGCCCAAGATAG	89
p21	TGCAAGAGAAAGCCCTGAAG	TGAATGAAGGCTAAGGCAGAA	96
p16^ink^	TTCTGGCAACGCGAGACTA	CTCCCTCCCTCTGCTAACCT	98
MnSOD	TGACTATGTAATGTTTTATCAGTTGGA	GTTGCTGACCACAGCCTTTT	91
CuZnSOD	TTGTGGTGTGATTGGGATTG	CAGTTTAGCAGGACAGCAGATG	80
ECSOD	ATCCCATAAGCCCCTAGCAT	ATTCGACCTCTGGGGGTAAG	84
Catalase	TTGGATCATGTCTTCCAAAAA	GGGAAAAGGAATCCGATCAA	83
Ku70	ACTGAGGGACATCTGCAAGG	TCCAAGTGTCTGCTGAGAGC	84
Ku80	GACATGAAGCTCTGGCCATC	TGTCTGTAGGGACCTGGAGTG	68
DNA-PKcs	CAACATTCGTGCACAAGAGC	CAAGGATGTTGGGGTCTGTC	92
myh-6	GAGCTGGAGAATGAGCTGGA	CTCTTCCTCATGCCCTTCAC	68
ppia	TGAGAACTTCATCCTGAAGCATACA	CATTTGTGTTTGGTCCAGCATT	89
Tfam	GTCTTGGGAAGAGCAAATGG	TTCACACTGCGACGGATG	70
